# Report on Certain Topics Connected with the Progress of Chemical Investigation

**Published:** 1876-06

**Authors:** Henry M. Lyman

**Affiliations:** Professor of Chemistry in Rush Medical College, Chicago


### REPORT ON CERTAIN TOPICS CONNECTED WITH THE PROGRESS OF CHEMICAL INVESTIGATION.

By HENRY M. LYMAN, M.D.,

PROFESSOR OF CHEMISTRY IN RUSH MEDICAL COLLEGE, CHICAGO.

(Read before the Chicago Medical Society, April, 1876.)

No very brilliant discoveries of a nature to captivate the popular fancy, have rewarded the recent labors of chemists. Something, however, has been accomplished in the way of utilizing previous discoveries, as may be seen by a recent paper on the preparation of oxygen, by Dr. A. W. Hoffmann, translated and published in *Nature*, Feb. 10 and 24, 1876. All the methods, usually noticed in text-books, for the preparation of oxygen, are passed in review, and a large number are described. The easiest and cheapest method for the production of the gas was originated and perfected by a French chemist, M. Tessié du Motay, who has, since 1867, experimented continually with the object in view to produce oxygen so abundant and cheap that it may be used in the arts as freely as we now use ordinary coal gas.

He has succeeded in preparing it in almost any quantity at a cost of three francs, or about sixty cents, gold, per 1,000 feet. The method consists in passing air over caustic soda and manganese di-oxide at a dark red heat. ($4\ Na\ OH + 2\ Mn\ O_2 + O_2 = 2\ Na_2\ Mn\ O_4 + 2\ H_2\ O$). The manganate of sodium, thus formed, under the influence of a dry current of overheated steam, disengages at the same temperature caustic soda, sesquioxide of manganese and free oxygen. ($2\ Na_2 Mn O_4 + 2\ H_2 O = 4\ Na\ OH + Mn_2 O_3 + O_3$).

The principal use of oxygen, thus far, has been in connection with the metallurgy of platinum. It has been recommended as an auxiliary for the Bessemer process of making steel—using a blast of oxygen instead of common air ; but the great difficulty of finding materials sufficiently refractory for the construction of the retorts and furnaces which would be needed, thus far hinders the adoption of such a method.

It is for illuminating purposes that the gas is now mostly employed. The oxyhydrogen lamp, employing zirconium-cones instead of chalk cylinders, yields a light sixteen and a half times as strong as the consumption of the same amount of coal-gas would afford. Various experiments for the illumination of streets and buildings by this method have recently been conducted in the European capitals. In Berlin it is said of the illumination of a railway station and its adjoining grounds, that "the effects caused by the little bluish flames are quite peculiar, and cannot be compared with any other light. The green of the trees seems more vivid, the colors of the dresses more brilliant, and above all, the faces of the people appear clearer—almost as distinctly as in full daylight."

Thus far, however, the necessity of duplicating every gas pipe, and the fact that coal gas must still be furnished, as the hydrogen element of the burning gas, so increases the expense and danger of this method of illumination that there is no immediate prospect that its adoption will become at all general.

In this connection I may be allowed to present a few estimates concerning the cost of illuminating gas manufactured in Chicago. It is difficult to get at the figures which are necessary to complete accuracy in such a computation, but sufficient data have in one way or another been made public to show that gas can be manufactured and sold for much less than is now charged for it, and still be made to yield a fair profit on the capital invested.

Cost of gas mains in Chicago, A. D. 1875:

| | |
|---|---|
| South Side | $1,818,680 |
| West Side | 1,256,640 |
| | 3,075,320 |
| To this must be added the value of the gas works, for which no figures have been published, but for which, with land, gasholders, and a stock of coal for a year, a very generous estimate would be | 2,000,000 |
| Cost of plant — total | $5,075,320 |

The cost of gas meters is not included, because they are virtually paid for by the gas consumers.

| | |
|---|---|
| The total amount of gas manufactured during the year 1875, was | 695,000,000 ft. |
| Deduct 10 per cent. for waste (a large estimate) | 69,500,000 |
| Leaves, for consumption | 625,500,000 ft. |
| The value of this amount of gas, at $2.50 per 1,000 feet | $1,563,750 |

| | | |
|---|---|---|
| The cost of producing this gas was, for wages of employes | $283,650 | |
| For price of coal, and expense of manufacture of 76,000 tons of coal | 395,777 | |
| Total cost of 625,500,000 feet of gas | | 679,427 |
| Total profit derived from sale of gas | | $884,323 |

(0.17.6 per cent. on $5,000,000.)

Besides this profit, every ton of coal should yield 170 lbs. of tar, estimated at 150 lbs. per ton. 76,000 tons 150 lbs. = 11,400,000 lbs. coal tar. Also, every ton of coal yields about 500 pounds of coke for sale = 76,000 × 500 = 38,000,000 pounds.

| | |
|---|---|
| Coal tar is worth about ½ cent. per pound—value of tar .................................... | $57,000 |
| Of coke there should be for sale about 1,140,000 bushels, worth at 8 cts. per bushel............ | 91,200 |

So that from the sale of coke and tar alone the income is sufficient to pay the taxes, and to make up for the wear and tear of the apparatus and gas mains (estimated at 15 to 20 cents per ton of coal used, each year.)

As a matter of fact, the gas companies, at the present rate of consumption, are sure of an income of not less than twenty per cent. on their investment, as long as the price of gas is held at $2.50 per 1,000 feet. It should be remembered, too, that there is no uncertainty or extra hazard about the investment. The manufacture is not liable to accident. There is no waste. Everything is saleable, and the exact amount of production can be calculated and anticipated with perfect accuracy. Money invested in the business is as safe as if it were put into Government bonds, and it is far more productive.

The recent improvements in the process of manufacture by which salicylic acid is obtained, are bringing that substance more largely into use. It differs from carbolic acid ($HC_6H_5O$) by the addition of the elements of carbonic anhydride ($HC_6H_5O + CO_2 = HC_7H_5O_3$).

Carbolic acid is obtained from coal tar, and as it may be converted into salicylic acid by anhydrous carbonic acid gas in the presence of an alkali metal, we have in this fact a new source of value for the coal tar which is produced in the distillation of illuminating gas.

Salicylic acid, when pure, is a white, crystallized substance, with a not disagreeable taste, and is soluble in three hundred parts of water, or in four parts of alcohol. It is not poisonous, for Prof. Traube prescribes it for acute rheumatism in doses of one gramme every hour until ten or fifteen grammes have been taken. Thus used it produces noises in the head, deafness, and antipyretic effects similar to quinine. It is, however, as a disinfectant, or antiseptic agent that it has been chiefly used.

It has been asserted that it is even more powerful than carbolic acid to destroy low organic forms and ferments. It is stated by Kolbe that its action is most decided upon those organic principles which, though formed in living tissues do not themselves constitute independent living forms. Such ferments are ptyalin, pepsin, emulsin, diastase, and similar unorganized causes of fermentative change. Upon the *organized* ferments, such as the yeast fungus and its class, salicylic acid seems to be more potent than carbolic acid. It also prevents putrefaction by destruction of those low forms of organized matter whose development in any given substance is the exciting cause of decomposition. This represents the current opinion regarding the substance. But in *Der Naturforscher*, for December, 1875, (quoted in *Nature*, Feb. 17, 1876, p. 317) is a paper by M. Fleck, of Dresden, "which appears to damp recent optimism in reference to salicylic acid as a means of disinfection. He finds that carbolic and salicylic acids may, under certain circumstances, even accelerate fermentation. Benzoic acid is more effective against fermentation, and cinnamic acid still better; but their small solubility in water is against their use. The antifermentative action of benzoic, carbolic and salicylic acids is dependent on the quantity of nitrogenous yeast-food; with increase of this the value of their action diminishes. The acids are not specific yeast poisons."

The recent use of nitrate of zinc, as a substitute for nitrate of silver, has been already noticed in the medical journals; and need not be mentioned further in this connection.

A French chemist, named Lecoq, has recently discovered a new metal resembling zinc. Delicately complimenting himself and his native country, he has named it Gallium.

The *American Journal of Science and Arts*, for January, 1876, contains an interesting paper by Prof. Draper, of New York, relative to the rotation of polarized light by solutions of quinine. He finds by experiment that

"the presence of sulphuric acid changes the rotation power of the alkaloid by 100°. Quinine used to be given in the form of sulphuric acid solution, and in the recently more popular form of pills or the like, its action is much less, and less certain; this difference being doubtless due to the change of molecular arrangement which is revealed in action of sulphate solutions of the alkaloid on light." (*Nature*, Feb. 17, 1876, p. 317.) Here, however, it may be questioned whether the professor has not too far left out of consideration the imperfect solubility and possible adulteration of many of the pills which are prescribed.

It is in the department of *physics* that are now being conducted those investigations which seem to promise most advantage to the progress of science. Mr. Crookes has recently performed a number of experiments which seem to prove that a ray of light falling upon a body *in vacuo* can communicate motion to a body so poised. The fact is disputed by some experimenters, who attribute the observed motion to the heat which may accompany light. However that may be, it is certainly a beautiful illustration of the power of *radiant energy* to produce motion in space.

Mr. J. Norman Lockyer is still pursuing his investigations with the spectroscope. He is gradually approaching the conclusion that the metallic elements represent forms of matter more simple than the *non*-metallic elements—in other words, that oxygen, chlorine, nitrogen, and elements of that class, are probably compound instead of simple substances.

H. C. Sorby, F.R.S., the well known president of the Royal Microscopical Society, has recently delivered a very interesting anniversary address on the *Relation between the limit of the powers of the microscope and the ultimate molecules of matter.** He shows that there is a "limit of visibility depending on the constitution of light." This "limit depends on the confusion in the image due to the

* *Nature*, Feb. 24, 1876, p. 332.

bright interference fringes overlapping the dark outlines of the object. This limit varies directly as the wave length of the light, and inversely as the sine of half the angle of the aperture of the object-glass when illuminated by means of a condenser of equal aperture." It is therefore necessary to believe that the normal limit of distinct visibility with the most perfect microscope is half of the wave-length of the light. If so, we must conclude that, even with the very best lenses, except under special conditions, light itself is of too coarse a nature to enable us to define objects less than $\frac{1}{80000}$ or $\frac{1}{100000}$ of an inch apart, according as a dry or an immersion lens is used. We must also conclude that *as far as this question is concerned*, our microscopes have already reached this limit."

Mr. Sorby then discusses the relation between this limit and the dimensions of the absolute atoms of matter as estimated by Stoney, Thomson, and Clerk Maxwell. A slight omission in his figures vitiates their absolute accuracy ; but is not sufficient to affect the conclusions drawn from their study. He shows that "in the length of $\frac{1}{80000}$ of an inch (the smallest interval that could be distinctly seen with the microscope) there would be about 2,000 molecules of liquid water lying end to end, or about 520 of albumen. Hence, in order to see the ultimate constitution of organic bodies, it would be necessary to use a magnifying power of from 500 to 2,000 times greater than those we now possess. These, however, for reasons already given, would be of no use unless the waves of light were some $\frac{1}{2000}$ part of the length they are, and our eyes and instruments correspondingly perfect. It will thus be seen that, even with our highest and best powers, we are about as far from seeing the ultimate structure of organic bodies as the naked eye is from seeing the smallest objects which our microscopes now reveal to us.

As an illustration, I have calculated that with our highest powers we are as far from seeing the ultimate molecules of organic substances as we should be from seeing the contents of a newspaper with the naked eye at the distance of a third of a mile; the larger and smaller types corresponding to the larger and smaller molecules of the organic and inorganic constituents."

According to this computation a mass of organic matter, the size of a blood-globule, $\frac{1}{3000}$ of an inch in diameter, would contain about 20,000,000,000,000 molecules; and "a spherical particle one-tenth the diameter of the smallest speck that could be clearly defined with our best and highest powers, might nevertheless contain no less than one million structural molecules."

The relation of these facts to the communication of disease through the agency of minute particles of organized matter is very obvious. When we reflect upon the fact that organized bodies may be traced to the very limit of vision without any indication that they constitute the primordial representatives of living matter, we are constrained to believe that below the limits of our vision there is still a world of organized forms—germs, some of them, of forms which may or may not expand into visibility—and that among these invisible entities may very probably be classed certain poisonous organisms, germinating under the influence of heat and moisture in particular soils, and impregnating the atmosphere with what we call *malaria*, as well as other organisms which, multiplying within the human body and conveyed thence to other human bodies, may occasion the phenomena of the various infectious or contagious diseases. If this be so, the microscope can only help our search for the causes of communicable diseases in the universe of visible objects, but can throw no light whatever upon the existence and nature of the lowest organisms. Referring once more to Mr. Sorby's computations, it is evident that every blood-globule in the body might be invaded by a living mass of organized matter in which were assembled no less than one million molecules—an army of Germans devastating France; and yet, before the commencement of its disintegration through the progress of the disease, the microscope could reveal no change whatever in the appearance of blood which had been thus entered. Nor need we be surprised at the virulence of such minute bodies. A drop of prussic acid may instantly kill a large animal. Why then should it seem strange that a germ which, though invisible, must be capable under favorable conditions of propagating its kind indefinitely, might so impregnate the tissues of an animal as to produce a dangerous if not fatal disease without any visible alteration of the histological elements of the tissues. We can only reach a knowledge of the existence and nature of such entities through the exercise of our reason upon the phenomena of disease, just as we arrive at a belief in the existence of interstellar ether through a consideration of the phenomena of light.

